# Supplementary material for: Mathematical model of the dynamics of transmission and control of sporotrichosis in domestic cats
Source: PLoS One. 2023 Feb 6;18(2):e0272672. doi: 10.1371/journal.pone.0272672 (PMC9901803; doi:10.1371/journal.pone.0272672)
Supplement: Attachment — Submitted filename: Response to reviewers.pdf [file pone.0272672.s001.pdf]

Response to reviewers:

Reviewer #1:

1) In page 4 the authors state that the probability of acquiring the infection is higher in females. Why?

In general, unneutered males are more affected by sporotrichosis due to behavioral habits. However, for this model, we assume that the probability of acquiring Sporothrix infection during mating is higher in females, because the bite of the dorsum of the neck, and the possibility of direct contact with exudate of cutaneous lesions in sick tom cats. Importantly, biting the skin of the neck is a remnant behavior used to immobilize the female and provide proper orientation for mounting.

We have added a shortened version of the above explanation, emphasizing that this probability is per contact. Males, however, have more risky contacts than females due to fighting behavior.

2) In section 3 the distribution of disease prevalence is obtained for the different groups considered in the model. Are there any empirical data on this for some comparison?

In section 3.1, the age-structure in the demographic equilibrium is calculated from demographic parameters obtained from the literature (fertility rate, and environment's carrying capacity) and from interviews with specialists (mortality rates by age class). No a priori guess is made about disease prevalence. All prevalences reported in figures 3 and 4 as well as in table 2, are the results of simulations under different scenarios until the system reaches its endemic equilibrium.

3) In the pdf, just after the figure for the flow chart of the model, there is a figure without any caption, which is repeated a few pages later. Please provide a caption and refer it in the text properly.

This has been fixed. We apologize for any confusion it may have caused.

4) For me the main shortcoming of the paper is the absence of a discussion of how the control of disease is implemented in the model. Is it turned on at some moment in time and set at some constant rate? Is it implemented just once? How do they change the model equations? Section 3.4 should be expanded with some discussion and details on this.

Multiple control scenarios are compared in the results (table 2) in terms of prevalence after 5 or 10 years of enforcement, Scenarios are combinations of pharmacological treatment of sick cats and Neutering at a pre-reproductive age. We have extended the description of the scenarios in section 3.4 to clarify the comparison of control scenarios.

Reviewer #2: Review

1) One of my main concern is regarding the mean field formulation of the model, because as the authors point out they took into account only domiciled cats (as opposed to stray cats), and therefore those cats might be highly clustered inside houses with very low interaction among them? Therefore the spatial distribution of cats might be highly relevant. Can the authors justify a bit more why the mean field approach is acceptable?

In this paper, we present a mathematical description of the dynamics of sporotrichosis transmission in a population of domestic cats, with street and neighborhood access, stratified by age and sex. The contact rates represent the average in the overall population. The mean field approach is justified because of the lack of the detailed characterization of the spatial clustering of cat populations. Moreover, the model also assumes that domiciled cats do have access to the street, which minimize the effects of clustering.

We have extended the first paragraph justifying the usage of mean field models given domestic cat's mobility and social contact patterns. We have added an extra reference about these behavioral characteristics.

2) Related with the above concern. It would be of importance to describe at some point in the manuscript, the behavior and area of movement of these domiciled cats. Can they circulate outside their houses through the neighborhood? If so, they might encounter other domestic or stray cats on the way. Those stray cats are not considered into the model...

Otherwise, where do the encounters occur among domiciled cats? In the same house? How many cats are on average in houses? I think that it's necessary to include in the manuscript a brief answer to these questions because they are relevant to disease transmission.

As explained in our response to the previous comment, we do assume that domestic cats have almost unrestricted access to the street population, which is

supported by the literature. Our study did not conduct a survey or census on any specific region, but we had conversations with colleagues that were experienced in such surveys that agreed with our assumptions.

3) page 4, in the model the probability of acquiring infection is assumed to be higher in females. Is there any reference the authors may cite to support this assumption? The same for the assumption that males have more sexual encounters than females. Please provide some citation for these assumptions.

In general, unneutered males are more affected by sporotrichosis due to behavioral habits. However, for this model, we assume that the probability of acquiring Sporothrix infection during mating is higher in females, because the bite of the dorsum of the neck, and the possibility of direct contact with exudate of cutaneous lesions in sick tom cats. Importantly, biting the skin of the neck is a remnant behavior used to immobilize the female and provide proper orientation for mounting. In relation to the assumption that males have more sexual encounters than females, under controlled conditions, one tomcat is usually sufficient for 20 females. In addition, during mating season, territorial males become increasingly irritable and protective of their areas. This is partly because other males wander great distances, with less recognition of territories, interacting with several groups of females. The increased contact between males can result in increased intermale aggression, particularly during encounters between individuals sharing an area. Intermale aggression, which is controlled by testosterone, can be violent and even take precedence over sexual behavior.

https://veteriankey.com/male-feline-sexual-behavior/#bib11

4) section 3.3 authors say from simulation results that: "In the absence of treatment, disease prevalence is greater among young cats, and among them in males." Is this result consistent with some field observation? Did the authors find evidence of this relationship in some reported study? If so, it can constitute an important piece of evidence supporting the model design.

In studies on feline sporotrichosis conducted in Rio de Janeiro, most of non-treated cats were male and young adults (median age was 24 months) (Reis et al., 2012; Souza et al., 2018; Boechat et al., 2018; Miranda et al., 2018).

We have added these 4 references to the text to support this statement.

5) Authors say that is important to treat cats when symptoms are appreciated. It would be important to mention in the manuscript which are those symptoms in cats, as well as in humans.

Feline sporotrichosis is clinically presented as a single lesion or as multiple cutaneous lesions (mainly nodules and ulcers). In addition, respiratory signs (eg.: sneezing, rhinorrhea and dyspnea) and lymph node enlargement are frequently observed. The most frequent clinical form is characterized by multiple cutaneous lesions with nasal mucosal involvement and respiratory signs (Gremião et al., 2021).

We have added this description to the Introduction for reference to interested readers.
